# Multipotent Mesenchymal Stem Cell Treatment for Discogenic Low Back Pain and Disc Degeneration

**DOI:** 10.1155/2016/3908389

**Published:** 2016-01-11

**Authors:** Jeffrey Zeckser, Michael Wolff, Jason Tucker, Josh Goodwin

**Affiliations:** ^1^Southwest Spine & Sports, 9913 N. 95th Street, Scottsdale, AZ 85258, USA; ^2^iOrthoBiologix, 12312 Copper Way, Charlotte, NC 28277, USA; ^3^Harborview Medical, 6151 Thorton Avenue, Suite 200, Des Moines, IA 50321, USA

## Abstract

Low back pain with resultant loss of function, decreased productivity, and high economic costs is burdensome for both the individual and the society. Evidence suggests that intervertebral disc pathology is a major contributor to spine-related pain and degeneration. When commonly used conservative therapies fail, traditional percutaneous or surgical options may be beneficial for pain relief but are suboptimal because of their inability to alter disc microenvironment catabolism, restore disc tissue, and/or preserve native spine biomechanics. Percutaneously injected Multipotent Mesenchymal Stem Cell (MSC) therapy has recently gained clinical interest for its potential to revolutionarily treat disc-generated (discogenic) pain and associated disc degeneration. Unlike previous therapies to date, MSCs may uniquely offer the ability to improve discogenic pain and provide more sustained improvement by reducing disc microenvironment catabolism and regenerating disc tissue. Consistent treatment success has the potential to create a paradigm shift with regards to the treatment of discogenic pain and disc degeneration.

## 1. Introduction

It is well documented that low back pain is a common and debilitating condition. Costs related to its medical expenses and lost wages exceed $100–200 billion annually in the United States alone. The intervertebral disc has been identified as the single most common pain generator for low back pain [1–3]. There is considerable interest in emerging bioregenerative therapies (specifically Mesenchymal Stem Cells) to treat painful pathologic discs as current interventional and surgical options appear to provide inconsistent pain relief and offer no restorative potential.

## 2. Intervertebral Disc Anatomy and Pathophysiology

When defining intervertebral disc (IVD) pathology, it is prudent to first briefly review normal disc anatomy and physiology. In simple terms, a disc is composed of the nucleus pulposus (NP), annulus fibrosis (AF), and the vertebral endplate. A remnant of the notochord, the NP contains randomly organized collagen and elastin fibers embedded in a highly hydrated gel-like matrix rich in proteoglycan (PG), which is synthesized by chondrocyte-like intervertebral disc (IVD) cells. The nuclear matrix is about 70–90% water, which is contained within the domains of proteoglycan. The NP plays an important role in spine flexibility and axial load distribution. The surrounding annulus (AF) is composed of parallel collagen fibers arranged in concentric lamellae (10–20 sheets). The cells of the AF are morphologically and phenotypically similar to fibroblasts. The annulus provides tensile strength and works in conjunction with the nucleus to absorb shock. The third component, the cartilaginous vertebral endplate, is composed of both hyaline and fibrocartilage and is intimately involved in connecting and anchoring the disc to the vertebral body [1, 4–6]. Innervation to the IVD is confined to the outer third of the annulus and endplate and is composed of microscopic nerve plexuses; the grey rami communicates anteriorly and the sinuvertebral nerves posteriorly [1, 4–6].

The IVD is a dynamic structure. The disc microenvironment appears to rely on a harmonious balance between anabolic and catabolic factors important for normal disc cell turnover. Growth factors including TGF-b, BMP, GDF-5, and IL-GF are examples of important anabolic factors. Conversely, catabolic metabolism is achieved via catabolic enzymes, inflammatory cytokines, proteinases, and aggrecanases. Examples include IL-1, TNF-alpha, ADAMTS, and MMPs. The disc is relatively avascular, consequently creating a harsh microenvironment (acidic pH, low oxygen tension, and paucity of nutrients) for IVD cells. Similar to most cartilaginous structures, this limited vascular support is a significant contributor to the poor natural regenerative capacity of the IVD when metabolic homeostasis is disrupted [1, 4, 6].

Clinical history and physical exam can be suggestive, but not diagnostic, of intervertebral disc pathology. Advanced imaging and diagnostics are needed for more accurate confirmation [1, 18]. MRI is the best noninvasive imaging modality that can be used initially to better define disc pathology [18, 19]. MRI can also be utilized to grade the severity of disc degeneration, detect vertebral endplate/subchondral bone pathology, and identify the presence of disc herniation [18, 20, 21]. In many instances, the presence of high intensity zones (HIZs) on MRI can be used to identify an annular disc tear [19]; however, MRI often lacks the ability to detect more centrally located fissures. Though controversial, provocation discography (without or without an analgesic component) may be employed as a means of both visualizing intradiscal pathology (via CT discography) and determining dynamically if the disc itself is the pain generator of interest (disc stimulation) [7, 22–24]. Using these imaging and diagnostic modalities, three related, but unique pathologic disc states can be described (Table 1).

Literature review reveals that these three pathologic disc states found in Table 1 are not consistently well understood and are often lumped together when describing discogenic pain [25]. It is of important to note that disc pathology often represents a continuum of disease where one form of disc pathology predisposes to another and where some degree of each may be found along with the primary pathology. Each pathologic state has unique characteristics and varies in its correlation with true discogenic pain, radicular pain, and predisposition to adjacent tissue irritation and degeneration [1]. To summarize, the primary pathology in IDD centers around painful fissuring of the annulus, though some nuclear degeneration also occurs. In DDD, nuclear degeneration predominates, compromising mechanical disc integrity and serving as a precursor to additional disc injury. Furthermore, a change in disc morphology leads to altered spine biomechanics may lead to irritation and degeneration of nearby tissues [1]. It is important to clarify that discogenic LBP may or may not be present in cases of DDD [1, 26, 27]. In disc herniation, acute radicular pain is most common, though axial LBP may occur if annular or somatic fibers are activated. Disc herniation may also alter disc integrity and predispose the disc to additional pathology over time [17].

## 3. Percutaneous and Surgical Interventions for Discogenic Low Back Pain

**Figure 1:**
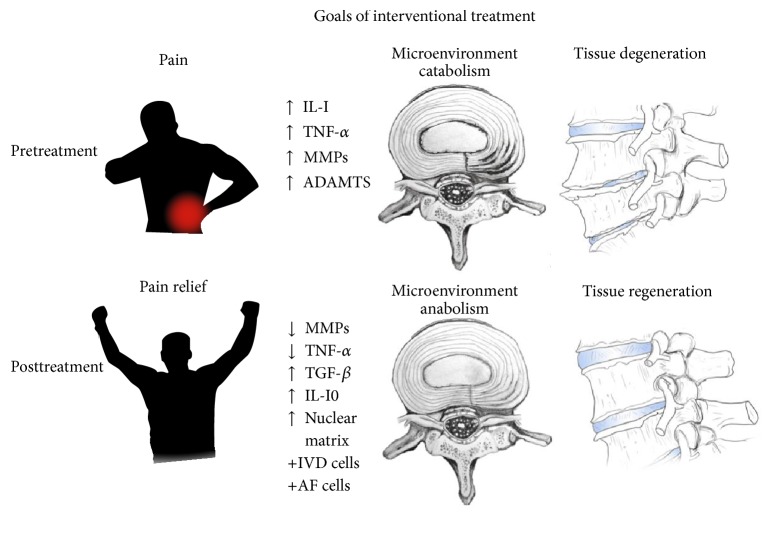
Goals of interventional treatment (pain relief, improved disc microenvironment, and tissue regeneration).

Along with a proper understanding of disc anatomy and pathophysiology, knowledge of currently available treatment modalities for discogenic LBP is important for enhancing patient outcomes and mitigating risks. If conservative treatment modalities fail, trained clinicians have traditionally used interventional techniques to aid in providing pain relief. However, we propose that interventional therapies should optimally strive to fulfill the following trio of treatment objectives: decline and/or resolution of primary nociceptive disc pain (with functional improvement), slowing and/or reversal of the catabolic metabolism (and associated degradation) within the disc microenvironment, and partial or complete restoration of disc tissue (Figure 1) [12, 28–30]. Readers are encouraged to evaluate available interventional treatment options to better understand their proposed mechanism of action, reported efficacy, and perceived disadvantages. One must also consider whether each modality possesses the ability to accomplish any, some, or all of the three stated objectives for the treatment of axial low back pain secondary to DDD, IDD, or disc herniation (Table 2).

Many of these therapies have shown some benefit (namely, in reducing pain); however, with the exception of a few listed emerging therapies, each are considered suboptimal in the treatment of discogenic pain because they fail to accomplish the entirety of the three previously stated objectives for disc pathology treatment. Most demonstrate evidence of short-term pain improvements, but many lack evidence of sustained benefit and/or complete relief. Though difficult to study, nearly all (with the exception of biologics) lack cellular support and consequently evidence demonstrating an ability to slow or reverse microenvironment catabolism, regenerate disc tissue, or restore native spine biomechanics. To achieve disc access, many of these interventions may promote a predisposition to further disc pathology with no ability to reverse this negative cascade [14, 31]. Furthermore, surgical modalities are considered a last resort for neurological compromise, instability, or intractable pain as they serve, namely, to reduce pain and improve stability via an essentially destructive process (tissue elimination or tissue fixation). This process alters native spine biomechanics and offers no restorative potential.

## 4. Multipotent Mesenchymal Stem Cell (MSC) Treatment

Traditional conservative, interventional, and surgical treatment modalities used in isolation or in combination have reported successes but are insufficient to accomplish the entirety of the three objectives outlined for the treatment of disc pathology. Percutaneously delivered Multipotent Mesenchymal Stem Cell (MSC) treatment has recently gained attention for its potential to revolutionize the treatment of discogenic LBP and associated disc degeneration. Contrary to traditional interventions, MSC therapy provides the necessary cellular support for regeneration and current research suggests that these treatments may have the ability to uniquely accomplish the three stated objectives for treating disc pathology (Figure 1).

It is important to first highlight key fundamental concepts of MSCs prior to discussing their clinical applicability in MSC-based intradiscal treatments. According to the International Society of Cellular Therapy, MSCs are defined by their ability to adhere to plastic under standard tissue culture conditions, express certain cell surface markers and lack expression of other markers, and differentiate into osteoblasts, adipocytes, and chondroblasts under in vitro conditions [74]. Inquiry has revealed that MSCs can act locally to accomplish many functions. In addition to displaying multipotency, MSCs are known for their self-renewal capacity as well as their chemotactic facilitation of nearby cellular activity. They are also able to modulate immunologic activities as well as exhibit trophic and anti-inflammatory effects on damaged tissues [75–78].

## 5. Mesenchymal Stem Cell Sourcing and Available Treatment Models

Currently, tissue engineers and clinicians appear most interested in the application of MSCs derived from bone marrow, adipose, and umbilical cord tissue. Synovium, skeletal muscle, and periosteum have also been rarely sourced [4]. Interestingly, no tissue source has shown clear superiority to date, with each displaying advantages and disadvantages. The most studied, MSCs derived from the bone marrow (BM-MSCs) appear well suited to both stimulate native disc cells and differentiate into IVD-NP cells. To obtain these cells, bone-marrow aspirate (BMA) can be collected (most commonly from the posterior iliac crest). BMA can then be centrifuged after harvest to obtain a nucleated cell concentrate referred to as Bone-Marrow Aspirate Concentrate (BMAc). If concentrated appropriately, a nucleated cell-rich concentrate including BM-MSCs along with other growth factors, mononucleated cells, and growth factor rich platelets can be generated. BM-MSCs have consistently shown ability to produce nuclear matrix componentry when cultured in a laboratory [4]. More importantly, BM-MSCs have been injected intradiscally in both animal (rat, porcine, canine, and sheep) and human models with promising results [79–86]. Disadvantages include a more cumbersome harvesting process and a decreased MSC density within the BM aspirate (compared to cells derived from adipose) [4, 82]. Adipose-derived MSCs (ADSCs) can be more easily collected from fatty tissue. Gene profile analysis has more recently demonstrated that ADSCs may be suited for easily acquiring a phenotype similar to that of IVD cells found in the nuclear matrix [27, 87]. Some consider adipose a superior source because of the relatively higher concentrations of MSCs within the tissue [26, 27, 78]. During the collection process, harvested fatty tissue is centrifuged to collect a layer known as lipoaspirate. Isolation of the Stromal Vascular Fraction (SVF) requires either enzymatic matrix digestion with collagenase or less desirable mechanical isolation [88, 89].

Human umbilical cord tissue-derived Mesenchymal Stem Cells (HUC-MSCs) may be easily collected from cord tissue and have potential allogeneic application. Perhaps the best source of cells for expansion, HUC-MSCs, could be well suited for allogeneic use because they appear to demonstrate low-immunogenicity [90]. There is data to support that HUC-MSCs can be beneficial for localized immunosuppression [91].

Researchers have identified other cell types worth mentioning that may work in concert with MSCs. There has been some interest in a niche of cells located within the disc referred to informally as mesenchymal progenitor cells. Recognition of these cells may be prove beneficial if they can be stimulated by exogenously administered MSCs or other biologics to aid in the regenerative process [27, 92–94]. However, they do appear to be suboptimal sources for cell harvest because they may be equally susceptible to degeneration, are markedly reduced with age, and require additional disc puncture to be extracted [27, 92, 93]. Lastly, some have explored using cells found at differing stages along the cell lineage such as juvenile or adult chondrocytes [95, 96]. Coric et al. demonstrated that injection of juvenile chondrocytes could improve pain, function, and disc morphology [95]. These more differentiated cells may lack key highlighted functions of MSCS but perhaps could be combined with MSCs for disc treatment and repair [27].

**Figure 2:**
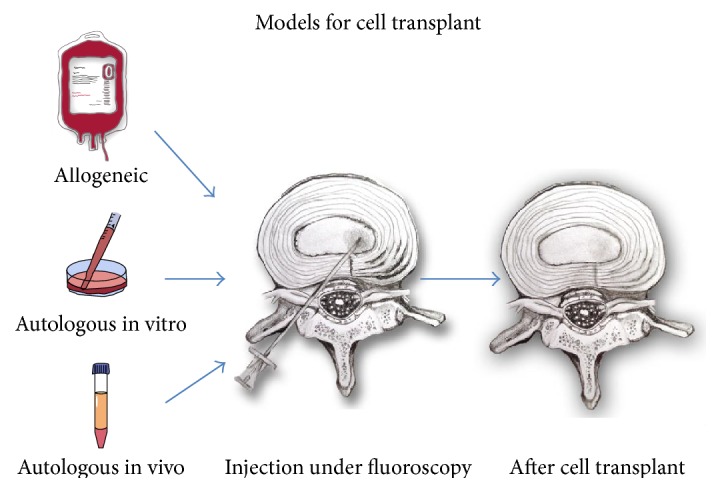
Models for mesenchymal stem cell (MSC) concentration and/or isolation prior to fluoroscopically-guided intradiscal injection.

Once harvested and isolated via centrifugation, MSCs (e.g., BM-MSCs, ADSCs) follow one of a few basic treatment modes/models prior to percutaneous cell implant (Figure 2). Cells can be directly reimplanted into the target tissue of the donor at the time (point-of-care) of extraction (autologous, in vivo model) or cultured outside the body and reimplanted into the donor at a later date (autologous, in vitro model) after purification and amplification [26, 27]. It is worth mentioning that cultured MSCs may additionally be combined with host tissue, a process termed ex vivo culturing [26, 27]. Significant advantages/disadvantages may exist with each of these models [4]. From a research standpoint, in vitro models (using animal or human cells) have been helpful in studying effects of varying cell (BM-MSC/ADSC) concentrations as well as how MSCs respond to progrowth stimulants. Using an in vitro model, MSC response (i.e., cell viability, differentiation) to manipulation of key factors (oxygen tension, nutrients, pH, osmolarity, cytokine levels, and exerted mechanical forces) can be assessed [4, 26, 27]. Clinically, in vitro models allow for cell expansion and differentiation prior to implant. Clinical application within the USA has been slowed in part by an FDA restriction against the use of cultured MSCs in humans. The FDA notes that transplant of human MSCs cultured in vitro constitutes more than “minimal manipulation” and falls under the same regulatory category as mass-produced drugs [88, 97].

In vivo models on the other hand are helpful for the study of safety, feasibility, and efficacy of MSC transfer. Several studies using an animal in vivo model have yielded valuable information about how injected cells perform within the disc environment [4]. The phenomenon of disc degeneration can be specifically studied in an animal in vivo model (via enzymatic digestion of disc tissue); however, this acute iatrogenic injury model is unable to precisely mimic DDD and to an even lesser degree simulate IDD [4]. Additionally, MSC effects using animal in vivo models are limited by differences between humans and animals (i.e., tissue size, cell populations, disc milieu, and spine biomechanics) [27]. Clinically, an in vivo model (“same-day” procedure) applied to humans is allowed per FDA and provides a “real world” scenario in which treatment effects such as pain and function can be assessed along with safety and tolerability of cell transfer. Additionally, quantification of tissue regeneration can be measured with MRI [4, 27]. Clinically applicable and less scrutinized, this model of MSC transplant is gaining traction as the method of choice in treating discogenic pain within the confines of the U.S.

A third basic cell transplant model exists in which cells may be isolated from a donor and transplanted into a separate recipient (allogeneic, in vitro model). Cells in this model may undergo in vitro culturing and/or may be simply stored without manipulation prior to future transplantation [94]. Unlike autologous cells, these cells may have an advantage of being transplanted “off the shelf.” Concerns of immune-reactivity (i.e., graft versus host) as well as questions about perceived efficacy have been raised [26, 98] (Figure 2).

## 6. Evidence for Mesenchymal Stem Cell Therapy in Discogenic LBP and Disc Degeneration

Bench-work research has provided positive evidence for the potential benefits of MSC transplant into pathologic discs. Disc microenvironment is important for growth and viability of native IVD cells as well as injected MSCs. In vitro studies combining MSCs and IVD cells have demonstrated bidirectional synergy promoting an anabolic environment. An in vitro study of combined rat IVD-NP cells and human synovial MSCs revealed suppression of genes related to matrix degradative enzymes and inflammatory cytokines [99]. Lui et al. showed evidence that BM-MSCs could secrete anti-inflammatory TGF-b and IL-10 [98]. The combination of MSCs and IVD-NP cells has resulted in IVD-NP cell proliferation and disc tissue regeneration [100, 101]. Both Richardson et al. and Strassburg et al. demonstrated the ability of human BM-MSCs to take on an NP-like phenotype as well as stimulating IVD-NP cells to produce new nuclear matrix componentry (cocultured system) [27, 101, 102]. Similarly, reciprocal effects were found when ADSCs were cocultured with IVD-NP cells [27, 103]. Though evidence is limited, notochordal (immature) IVD cells as well as IVD-AF cells have also been cocultured with MSCs with promising results [4, 104, 105]. Of interest, Gebraad et al. recently demonstrated that human ADSCs took on an AF phenotype when cultured in serum-free chondrogenic media [106]. Combination treatments with additional biologics (e.g., growth factors) may prove valuable for preconditioning, growth, differentiation, and maintenance of MSCs but require further investigation [4, 27, 107].

The application of MSCs in various in vivo animal models has demonstrated similar anticatabolic and disc-tissue regenerative effects. In postnucleotomy rabbit models, injection of BM-MSCs has demonstrated suppression of nuclear collagen type 1 formation (causing fibrosis) as well as restoration of disc height values and MRI signal intensities approaching 81% and 91%, as compared to sham-treated discs at 67% and 60%, respectively [108, 109].

Using a canine in vivo model, Hiyama et al. similarly demonstrated that an injection of BM-MSCs into the nucleus of enzymatically degenerated (postnucleotomy) discs showed increased proteoglycan (PG) content consistent with normal nondegenerated discs at 12 weeks posttreatment; control postnucleotomy discs showed marked decrease in PG [83, 109]. A more recent study was performed in sheep using allogeneic Immunoselected-STRO-3+ Mesenchymal Precursor Cells (MPCs) (antibody selected). Adjacent enzymatically degenerated discs were injected with either low dose MPC or high dose MPCs (within a hyaluronic acid (HA) carrier). A third degenerated level was injected with HA-alone (control). Combined histopathology scoring showed statistically significant higher scores in low dose MPC (at 3 months) and high dose MPC (at 6 months) when compared to internal and external controls. Although all three injected discs showed some disc height recovery at 6 months, only those injected with MPCs (low and high dose) showed statistically equivalent MRI scores to that of nondegenerated controls [79].

Since there are clear mechanical differences on the IVD with a quadrupedal animal, human in vivo studies are needed to confirm reduction or elimination of discogenic pain. A pilot investigation by Orozco et al. using in vitro expanded autologous BM-MSCs has demonstrated early safety and feasibility in a percutaneous approach. Ten patients with persistent discogenic LBP, DDD (>50% disc height loss), and an intact outer annulus (per discography) underwent a single injection after failing 6 months of conservative treatment. The authors reported statistically significant improvements in both pain and disability after 3 months, with results persisting at one year. Although there was no recovery of disc height on MRI, water content of the discs significantly improved at one year [110]. In a recent pilot study by Pettine et al., twenty-six patients with discogenic LBP (DDD-Pfirrmann grades IV–VII) received percutaneously injected BMAC (same-day procedure). In 8 patients, provocative discography was used to confirm discogenic etiology. Authors found a reduction in ODI and VAS scores from 56.5 and 79.3 at baseline to 22.8 and 29.2 at 3 months, which continued throughout 12 months. Additionally, eight of twenty patients improved by one modified Pfirrmann grade (on MRI) at one year. Interestingly, authors found that patients receiving greater than 2,000 CFU/mL of MSCs experienced significantly faster and greater reductions in VAS and ODI scores [2], justifying the need to concentrate BMA in a same-day procedure. The authors of this review are currently enrolling 30 patients for a prospective, blinded, RCT investigating safety and preliminary efficacy following an injection of autologous BMAc (same-day procedure). Additionally, a prospective, multicenter, double-blinded RCT performed in humans is currently being performed to evaluate safety and preliminary efficacy of allogeneic MSCs combined with an HA carrier in 100 patients with clinically determined single level disc pain and degeneration (Pfirrmann III–VI). Patients were randomized to receive a single injection of proprietary allogeneic low or high-dose MSCs + HA, HA-alone, or saline (control) and are followed for a 2-year period. After 1-year, 69% of the low-dose MSC and 62% of the high-dose MSC groups have showed greater than 50% reduction in VAS scores compared to 35% and 31% in HA-alone and saline groups. A mean reduction in ODI of 43% (high-dose MPC), 35% (low-dose MPC), 30% (HA-only), and 28% (saline) has been reported [111]. Results of data analysis at 24 months was recently announced which demonstrates that 46% (after 12 months) and 48% (after 24 months) of the low-dose MPC group achieved minimal or no residual pain (VAS < or = 20) compared to 13% of saline-treated patients [3].

These cited studies have added to the growing body of evidence suggesting that percutaneously injected MSCs may uniquely provide clinical improvements in discogenic pain, reduced microenvironment catabolism, and reduced disc tissue loss. In vitro studies show evidence that MSCs promote anti-inflammatory and anticatabolic effects within the disc microenvironment. Furthermore, in vitro and in vivo studies (mostly in animals) have demonstrated MSC and IVD cell proliferation, nuclear matrix production, improved annular integrity, and recovered disc height. In vitro culturing of human MSCs has shown many of these same effects. Though limited clinical studies have been conducted to date, improvements in pain and function following intradiscal MSC injection have been demonstrated. Evidence supporting disc tissue regeneration exists and there is evidence now demonstrating at least partial recovery of disc tissue in both animals and humans, [2, 79, 110, 112]. Though limited clinical studies have been performed to date, improvements in pain and function following MSC injection have been demonstrated in addition to safety and feasibility. Further human studies are required to further support the initially observed potential benefits.

## 7. Cell Scaffolds as an Adjunct to Mesenchymal Stem Cell Therapies

The efficacy of intradiscally injected MSC based treatments may be potentially enhanced by combination therapies. The concept of an added cell matrix or scaffold has been introduced and has been shown to aid in cell delivery, orientation, differentiation, growth, replication, cellular metabolism, and sustained viability. Naturally occurring biopolymers can be used alone or in combination as hydrogel scaffolds for in vitro and in vivo MSC preparation and treatment. Examples include collagen-type I and solubilized atelocollagen, calcium alginate, chitosan, KLD-12 peptide, carboxymethyl cellulose, and collagen-type II. Hyaluronan-based hydrogels have been frequently used as a scaffold for MSCs with noted success in rat, sheep, and more recently human models [79, 86, 110, 111]. Another biopolymer, fibrin continues to show genuine promise as a presealant or scaffold for MSC therapy because of its sealant properties and anti-inflammatory and promatrix effects [49, 113]. Each of these biopolymers conveys certain advantages, but none has proven clearly superior to date. Numerous synthetic polymers have also been created to optimize the most desired scaffold characteristics [7, 13, 27]. Given the structural differences found within the nucleus and annulus, different combinations of biopolymers and synthetic polymers are currently being considered [13, 114]. These polymers may be permanent; however, most polymeric scaffolds of interest are either thermoreversible or susceptible to enzymatic degradation. This may be ideal for allowing needed space for regenerated tissue growth [7, 13, 27].

## 8. Safety Concerns with Mesenchymal Stem Cell Therapies

The clinical application of intradiscally injected MSCs has raised potential concerns. Chief among these is the potential for MSC-promoted carcinogenesis. It is well documented that embryonic stem cells (found earlier in cell lineage) can promote tumor formation [115]. However, multipotent MSCs are present at a more committed cell stage and have a more limited differentiation potential than totipotent embryonic stem cells. With regards to in vitro culturing, there is some concern that increased cell passage of MSCs may increase risk for spontaneous cell mutation. This concern should not exist with autologous, same-day procedures in which cells are not altered or cultured but exist as they were in the body previously. To date, there has been no published evidence demonstrating carcinogenesis of multipotent MSCs using in vivo or in vitro models [94, 115]. Concern for abnormal tissue formation exists. A study by Vadalà et al. demonstrated ectopic osteophyte tracts in a rabbit model in which BM-MSCs were found extravasating beyond the margins of the outer annulus [116]. It has been demonstrated that added care must be taken when combinations are added to augment MSC efficacy. Chen et al. demonstrated evidence of intradiscal osteogenesis promotion with combined MSC and PRP injection in an ex vivo porcine model; however, this too has not been reported in humans [117]. Adding further complexity, growth factor effects of certain biologics have been shown to be pleiotropic (producing multiple effects) at varied concentrations [118]. Though evidence is lacking, there is potential that promotion of angiogenic factors may enhance nerve and vessel ingrowth (from peripheral annulus towards nucleus) [119–121]. Aside from safety concerns, some question MSC sustainability given the harsh milieu of the disc. Axial and torsional forces that are hard to mimic in animal models may hamper MSC efficacy in human discs as well [107, 122].

## 9. Conclusion

Percutaneous injection of MSCs into the intervertebral disc may uniquely fulfill the objectives of treating disc pathology with clinical improvements in pain and decreased microenvironment catabolism. Some evidence suggests that MSCs may fulfill the final major objective of reversing disc tissue loss. At this time, MSC-based therapies appear most suited for the treatment of discogenic pain secondary to IDD and DDD, serving as an obviation of more invasive surgical interventions. If successfully accomplished early in the disease process, this proactive approach may aid in preserving native spine biomechanics, ideally resulting in less degeneration of the IVD and surrounding tissues structures and again serving as an obviation to future surgical interventions. As the therapy advances, there may be some role for these therapies in improving the disc microenvironment and structural integrity in cases of small disc herniations; some have suggested a similar role in postsurgical discs (i.e., partial discectomy) [4, 17, 96, 123]. Proper patient selection will be important as MSC based therapies should not be thought of as a “cure all” for spine pain. Realistically, patients with non-IVD pain generators as well as those with advanced disc degeneration or severe annular compromise may not be ideal candidates for this therapy.

A continued collaboration between researchers and physicians will be important in optimizing the therapeutic potential of MSC-based therapies as well as the refinement of optimal MSC cell type, concentration, and supportive componentry. As the field advances, factors unique to both autologous and allogeneic cell preparations should be further studied and considered with each having advantages and disadvantages. Ideally, in vitro cell culturing should be compared to same-day therapies in terms of safety and efficacy. Given the harsh environment of the disc (acidic pH, low oxygen tension, and paucity of nutrients) development of future therapies may include preconditioning cells prior to transplant (e.g., genetic manipulation and cell culturing in harsh conditions) to enhance survivability. Additionally, studies are needed to determine an optimal time course for applying MSC treatments. Traditionally, symptomatic patients have been encouraged to pursue conservative therapies, reserving interventional injections for nonresponders and spine surgery for severe, refractory axial pain. This dogma has existed in large part because previous interventional and surgical options have inconsistently provided pain relief and have not offered the same restorative potential that may be present with MSC-based treatments. If demonstrated consistently safe and effective, MSC-based treatment may lead to a paradigm shift towards more aggressive, nonsurgical care for patients with discogenic LBP (IDD/DDD) and other forms of compromised disc integrity.

## Figures and Tables

**Table 1:** Intervertebral disc pathology.

Internal disc disruption (IDD)	(i) Defined by the development of focal fissures extending outward from the nucleus into the annulus (radial fissure) or along annular lamellae (circumferential fissure) [1, 7].
	(ii) Annular fissures provide a conduit for inflammatory chemical mediators to trigger nociceptive nerve endings in the outer AF [1, 7]. Additionally, “nerve ingrowth” along fissure sites can increase exposure to nociceptive and mechanical stimuli [1, 7]
	(iii) Discogenic pain may develop when annular pain fibers are directly stimulated by inflammatory mediators or are indirectly stimulated secondary to increased mechanical loading pressures [1, 4, 6].
	(iv) Though nuclear degeneration is minimal in early stages of IDD, it is believed to trigger a catabolic cascade within the microenvironment of the disc, which serves as a precursor to overt disc herniation and DDD at later stages [1, 7].
	(v) IDD is considered to be the most common detectable cause of LBP (estimated prevalence of 39%) [1, 8].

Degenerative disc disease (DDD)	(i) Defined as a diffuse, progressive, and age-related phenomenon defined by nuclear dehydration and fibrosis and resultant disc space narrowing (3-4% loss per year) [1, 4, 9, 10].
	(ii) Mechanical, biochemical, nutritional, and genetic factors contribute to a shift towards catabolic metabolism within the disc microenvironment. Hallmarks include increased metalloproteinase (MMP) activation, decreased IVD cell viability, and decreased proteoglycan (PG) production [4, 9, 11, 12].
	(iii) Resultant increased disc space narrowing can cause a redistribution of axial mechanical forces on nearby structures (e.g., vertebral body endplates, facet joints) causing tissue irritation and degeneration (i.e. osteophytes, buckling) [1, 13].
	(iv) DDD may or may not result in discogenic LBP but almost universally compromises disc integrity, predisposing the disc to further injury [4, 12, 13].

Disc herniation	(i) Defined by a displacement of nuclear disc material beyond the normal contours of the outer nucleus [1, 5].
	(ii) Stages include bulge, protrusion, extrusion, and sequestration
	(iii) It is the most common etiology of radicular leg pain, via chemical radiculitis or mechanical compression of nerve roots [1].
	(iv) May contribute to focal LBP as a result of inflamed dura of a surrounding nerve root sleeve (somatic referred pain) or from activation of outer annular pain fibers within the injured disc [1].
	(v) Data suggests an alteration of the annulus may contribute to decreased disc integrity and accelerated DDD [13–17].

**Table 2:** Interventional and surgical treatment strategies for discogenic low back pain.

Intervention	Mechanism of action, reported efficacy, disadvantages
Percutaneous epidural steroid injections	Steroids (injected into the epidural space) are thought to create a local chemical effect with suppression of inflammatory mediators and/or stabilization of nerve membranes [32–35]. Evidence for epidurals for radicular pain relief is good; however, two more recent systematic reviews by Manchikanti and Benyamin conclude that evidence is variable, insufficient, and fair at best for the use of epidurals in treating persistent discogenic LBP [36–41].
Percutaneous intradiscal injections	
(A) Steroids	(A) Steroids (injected into the disc) are thought to create a local chemical effect within the disc. Conflicting evidence has been reported. Most influential, a double-blinded RCT by Khot and colleagues failed to demonstrate efficacy for steroid use within the disc [42]. Potential concern for chondrotoxicity exists given the chondrocyte-like nature of IVD cells [43, 44].
(B) Neurolytics	(B) Neurolytics result in cessation of nerve signal via nerve-lysis. An RCT by Peng et al. showed substantial improvements in pain and disability following intradiscal methylene blue injection [45]. Kim et al. showed significant improvements in pain and function; however, improvements were not consistently sustained past 3 months [46]. Other agents such as chondroitin sulfate, glucosamine, carboxycellulose, and a cephalosporin antibiotic have also been injected showing at most modest efficacy [28].
(C) Coagulation	(C) Intradiscal Electrothermal Therapy (IDET) serves to coagulate nerve fibers at targeted radial fissures. There has been lack of consistent evidence to support IDET, with reports citing 40–90% success for achieving at least 50% relief; consequently the therapy has fallen out of favor. In need of more rigorous study, transdiscal biaculoplasty uses a radiofrequency current to accomplish this same goal of coagulation (with simultaneous cooling) [47]. The Ramus Communicans nerve fibers can also be ablated. Malik recently noted evidence of short-term efficacy for Ramus Communicans Ablation [25].
(D) Fibrin sealant	(D) Fibrin seeks to treat discogenic pain by sealing painful annular fissures. Some evidence suggests fibrin may help to downregulate microenvironment catabolism, seal annular fissures, and increase disc height [48]. When used in combination it may also serve as a tissue scaffold for cell-based therapies [12, 48]. Though an initial pilot study provided promising results, a more recent multicenter, blinded RCT failed to demonstrate similar efficacy following intradiscal injection of “off the shelf” fibrin producing products [49–51]. In need of further study, autologous fibrin preparations are also being explored.
(E) Prolotherapy	(E) Dextrose prolotherapy is proposed to produce chemomodulatory effects and promote tissue repair through stimulation of inflammatory and proliferative phases [52, 53]. Intradiscal injection of dextrose has demonstrated improvements in radicular leg pain; however, improvements in axial LBP have not been assessed [52]. Traditionally, entheses sites have been targeted (not within the disc) for the treatment of nonspecific LBP. A systematic review using 5 RCTs and quasi-RCTs found mixed results in terms of pain and disability following prolotherapy in LBP patients [53]. Like other proinflammatory injections, prolotherapy can result in a postinjection painful flare [52].
(F) Other biologics	(F) Growth factors (GFs) and platelet-based therapies (platelet-rich plasma (PRP), platelet lysate) are believed to have the ability to desensitize cutaneous nerve endings, decrease tissue catabolism, and promote tissue regeneration. Synergism with resident progenitor cells may result in antinociceptive effects and encourage the proliferation of IVD cells, nuclear matrix, and annular collagen [54–57]. Solitary growth factors appear to differ in their anti-inflammatory properties as well as their ability to induce IVD cell activation and matrix proliferation [55, 58]. Members of the TGF-b superfamily have shown greatest promise; however, other potentials include BMPs, IGF-1, GDFs, EGF, PDGF, and bFGF [55]. Animal studies have provided evidence of decreased inflammatory cells and improved fluid content and disc height in discs injected with PRP [58, 59]. Additionally, PRP injected sooner after disc injury appears superior to injection at a later time [59]. Results of a prospective, double-blinded RCT in humans has yielded statistically significant reductions in both pain and function at one year [60, 61]. Anecdotally, some have chosen to inject these biologics outside of the disc in the epidural space to downregulate somatic fibers and induce disc healing.
Surgical	
(A) Decompression	(A) Disc decompression aims to treat discogenic and radicular pain by decompressing herniated disc tissue through tissue removal or ablation [47]. Microdiscectomy, automated percutaneous decompression, laser discectomy, and disc nucleoplasty have been used. Decompression is often used for patients with disc herniation and acute neurologic decline; however, others make use of it in cases of refractory radicular and/or axial pain. A recent systematic review concluded there is limited to fair evidence supporting the use of nucleoplasty for radicular pain [62]. It was shown to be moderately superior to nonsurgical therapy for improvement in back pain in the first 2-3 months; however, it was not statistically superior at 2 years [25]. Other reviews have concluded there is no evidence for its use in cases of isolated axial LBP [62–64]. Laser decompression has gained recent attention, with individual studies reporting good outcomes; however, a systematic review by Singh et al. concluded there is limited evidence to support the use of laser therapy [65, 66]. Collectively, these therapies remove disc tissue and should be utilized prudently because of the risk of accelerated disc degeneration [16].
(B) Arthrodesis	(B) Arthodesis involves fusing adjacent vertebral bodies for stability. Mechanism of fusion (osseous, hardware) and level of invasiveness (percutaneous, arthroscopic, laparoscopic, and open) vary. One randomized study reported excellent or good outcomes at 2 years in 46% of surgical patients versus 18% nonsurgical patients [67]. More recently, a review of four high quality studies concluded that fusion is slightly moderately more efficacious than standard nonsurgical therapy, but no better than intensive rehabilitation in terms of pain and function [68]. Reduced spinal mobility and a change in spine mechanics is believed to contribute to pain recurrence and an increased incidence of adjacent disc segment degeneration (ASD) (23–43%). Risks of infection, epidural fibrosis, postlaminectomy syndrome, and hardware malfunction have also been reported [8, 27, 69, 70].
(C) Arthroplasty	(C) Arthroplasty involves substituting native discs with artificial discs. Arthroplasty may be superior to bony fusion with respect to maintaining basic motion and spine mechanics. Prevalence of ASD is reportedly 9% and 6.7% after arthroplasty (compared to 34% and 23.8% for fusion) [69, 70]. A recent systematic review, Jacobs et al. concluded there was no evidence of superiority between disc replacement and fusion surgery [71, 72]. Risks include hardware infection as well as spinal cord damage and nearby tissue inflammation following hardware degeneration [27, 73]
